# Supplementary material for: South Africa’s male homicide epidemic hiding in plain sight: Exploring sex differences and patterns in homicide risk in a retrospective descriptive study of postmortem investigations
Source: PLOS Glob Public Health. 2023 Nov 22;3(11):e0002595. doi: 10.1371/journal.pgph.0002595 (PMC10664949; doi:10.1371/journal.pgph.0002595)
Supplement: Attachment — Submitted filename: response to reviewers.docx [file pgph.0002595.s005.docx]

Dear Editors

Thank you so much for the helpful comments and queries that have helped to improve our manuscript as well as encouraging us to submit a revision.

As per your instructions, we have uploaded a revised manusript with tracked changes and an unmarked version, as well as the PloS questionnaire on inclusivity, and responded to each of the comments raised by the editorial team and the three reviewers, which we have set out in the bulleted list that follows.

**Comments from the editors:**

1. A rebuttal letter that responds to each point raised by the editor and reviewer(s). You should upload this letter as a separate file labeled 'Response to Reviewers'.

A marked-up copy of your manuscript that highlights changes made to the original version. You should upload this as a separate file labeled 'Revised Manuscript with Track Changes'.

An unmarked version of your revised paper without tracked changes. You should upload this as a separate file labeled 'Manuscript'.

Our response:

As per the current letter.

2. Please include a complete copy of PLOS’ questionnaire on inclusivity in global research in your revised manuscript. Our policy for research in this area aims to improve transparency in the reporting of research performed outside of researchers’ own country or community. The policy applies to researchers who have travelled to a different country to conduct research, research with Indigenous populations or their lands, and research on cultural artefacts. The questionnaire can also be requested at the journal’s discretion for any other submissions, even if these conditions are not met.  Please find more information on the policy and a link to download a blank copy of the questionnaire here: [https://journals.plos.org/globalpublichealth/s/best-practices-in-research-reporting](https://protect-za.mimecast.com/s/IgbACKO7nQI2Bz1WJfvEboS). Please upload a completed version of your questionnaire as Supporting Information when you resubmit your manuscript.

Our response:

This has been included with the current submission.

3. Please send a completed 'Competing Interests' statement, including any COIs declared by your co-authors. If you have no competing interests to declare, please state "The authors have declared that no competing interests exist". Otherwise please declare all competing interests beginning with twhe statement "I have read the journal's policy and the authors of this manuscript have the following competing interests:"

Our response:

We have aupdated our CoI statement as follows:

“The authors declare that the research was conducted in the absence of any commercial or financial relationships that could be construed as a potential conflict of interest. BB serves on the board of non-governmental organisation, Gun Free South Africa, but receive no remuneration for this role.”

4. We noticed that you used "data not shown" in the manuscript. We do not allow these references, as the PLOS data access policy requires that all data be either published with the manuscript or made available in a publicly accessible database. Please amend the supplementary material to include the referenced data or remove the references.

Our response:

The epidemiology of all injury mortality has been comprehensively covered in a technical report we cite as a source. We have moved information about the sample realisation and total number of injury cases to the section on sampling in methods, where we have cited the original technical report. This report contains all the necessary information for readers to explore the broader injury profile in South Africa.

5. We do not publish any copyright or trademark symbols that usually accompany proprietary names, eg  ©, ®, ™  (e.g. next to drug or reagent names). Please remove all instances of trademark/copyright symbols throughout the text, including © on page 11.

Our response:

We have changed the footnote.

6. Methods: The sampling methods and case selection criteria are clearly described. However, it would be helpful to state the final sample size included in the analyses after exclusions.

Our response:

There were no exclusions from the sample per se, but a categorisation of data into different causes of death of which homicide was a specific category. As described in our response to question 4 this has been comprehensively covered in the technical report that we cite as the source in *Methods: Sampling.*

7. Methods: Were any steps taken to address missing data? This is not discussed but seems relevant for variables like blood alcohol concentration.

Our response:

There was a negligible amount of missing data except for the BAC (alcohol) variable and so no adjustment has been made in the descriptive data (Tables 1 and 2) for the temporal variables (day and month) and age, for which missing data accounted for <1% and <3% of all cases respectively. In the methods section (statistical analysis), we reported that we applied a scaling factor for age data in our calculation of rates.

The high level of missing data for alcohol excluded the possibility of adjusting or imputing data. We have added the following sentence to the limitations section in the discussion suggesting that these data be interpreted with caution: ”The large number of cases missing blood alcohol data (83% of all cases) requires that these findings be interpreted with caution.”

We have also removed the finding about the difference in alcohol levels between males and females from the abstract and added a further footnote to table 1.

8. Results: The results are comprehensive and informative. The tables effectively summarize key findings.

Our response:

Noted with thanks.

9. Results: It would be interesting to see age-stratified rates for `. Are the differences most pronounced in certain age groups?

Our response:

We agree that this further analysis might be considered interesting, but our review of the data suggests otherwise. Note that the technical report for the overall study already includes some preliminary analysis comparing firearm versus non-firearm (i.e. all other mechanisms combined) by age and sex. These data indicate that 1. firearms account for a higher proportion of deaths among men for all age strata. 2. There is limited fluctuation by age amongst males aged 5-69 years and 80+ years (29-41% of homicides) and women aged 5-79 years (22-33% of homicides). The only outliers were males aged 70-79 (+-22% of deaths) and females 80+ (8%) and both of these age categories account for very few cases.

While it may well be possible to present these data and also other causes (sharp, blunt, strangulation, etc) the write up and discussion of these additional results might become cumbersome. We were also concerned at the already lengthy descriptive tables that we have provided and how we might justify selective stratified analysis of some variables (e.g. age x sex x cause) instead of others (age x sex x province), (age x province x cause), etc.

So we have elected not to present this additional stratification in our revision, but can certainly revisit this if our rationale is not supported.

10. Discussion: The discussion contextualizes the results well and makes a strong case for the need to address violence against men and boys.

Our response:

Noted with thanks.

11. Discussion: When comparing to the previous 2009 national study, it would be helpful to comment on whether the sampling methods were comparable between the two time points.

Our response:

We have clarified this in *Methods: Statistical analysis* as follows:

“We used generalised linear models and linear regression models to assess the association between sex and victim characteristics including age and mechanism of injury (guns, stabs and blunt force) within and between the current survey (2017) and the 2009 study,(2) which used sampling methods that were comparable at a national level..”

12. Discussion: Were statistical tests done to assess changes over time? If so, it would be useful to report whether observed differences were statistically significant.

Our response:

The comparison between just two time periods limits our opportunity for performing temporal analyses with time/year as a continuous variable and instead we perform a categorical analysis comparing the two time periods (2009 and 2017) in Table 3.

Nevertheless we have edited text in the first paragraph of discussion citing the 2009 study and clarifying that the overall homicide rate decreased among both men and women.

13. Discussion: Overall this is a nicely written paper that makes an important contribution to the literature on violence and injuries in South Africa. My suggestions are minor and intended to provide a bit more methodological detail.

Our response:

Noted with thanks.

**Reviewer 1 comments**

14. Why is data from only 8 out of 9 provinces were collected?

Our response:

Data were collected from all nine provinces. This has now been clarified in the methods. We have also corrected the number of Western Cape mortuaries from 18 to 16. Two of the smaller mortuaries were closed in 2015/16 and their caseloads consolidated with larger regional mortuaries.

15. Briefly discuss about alcohol test. what was teh test, how, when because there are likelihood of error and bias.. since it is mentioned as (1) objective in the introduction section.

Our response:

Thank you for noting that omission in *Methods: Case selection and variables*. We have included blood alcohol concentration as one of the variables collected from the postmortem report and note that it was analysed using gas chromatography with flame ionization detection method.

Also, we noted the PloS guidelines on categorizing humans by race/ethnicity, age, disease/disabilities, religion, sex/gender, sexual orientation, or other socially constructed groupings and included a qualifier explaining that “sex was inferred from the biological sex recorded in the postmortem report”.

**Reviewer 2 comments**

16. What proportion of homicides were due to multiple mechanisms (blunt and penetrating for example)?

Our response:

We have added a footnote to Table 1 as follows:

“Postmortem reports specified a primary cause of death for each death, which we assigned as the external cause. In addition, we noted 2833 cases with multiple injuries, of which 2426 were male, 405 female and 2 unknown.”

17. There are a few typos on page 5 – “homides” instead of “homicides”; missing word in the second sentence in the Discussion section. Another round of copyedits will suffice.

Our response:

We apologise for these errors and have proofed the document carefully.

**Reviewer 3 comments**

18. While the data represents a very interesting source of information, the conclusions need to be considerably refocused. The comparison between homicide in men and women while justified in theory by the data, does not really provide any helpful information that should educate future interventions.

Our response:

We agree that the data justify a comparison between men and women, but also believe that the comparison serves to highlight a critical, neglected public-health priority. That is one of the key conclusions of the paper, supported by the empirical data that we have presented.

19. The focus on gender is a missed opportunity in terms of use of this data, and this paper would benefit from being focused more on the high rates of homicide in men and specific sources of homicide rooted in geography, sociodemographic characteristics other than gender, and a more data-driven conclusion.

Our response:

The paper does indeed focus on the high rates of homicide in men. We have provided descriptive data for all available variables of interest, but must clarify that the study is limited by the data that are recorded and made available through the post mortem investigation process.

We have also made it very clear that prevention of male violence should not be prioritized over violence against women and children, and that violence prevention should be *inclusive* as set out in Sustainable Development Goal Target 16.1. Furthermore, to emphasise the importance of considering violence prevention for males alongside, and not at the expense of, females, we cite local burden of disease research (Prinsloo et al, 2022) that indicates the comparable burdens when the full range of health impacts are considered.

20. The authors seem to interject a lot of opinion in the study regarding poverty, inequality, firearm use, and alcohol use, but this is not really discussed in full detail in the study because of the focus on gender.

Our response:

Poverty, inequality, firearm and alcohol use are well-known risk factors for violence, and highly prevalent in South Africa. We have cited relevant supporting literature. An in-depth exploration of these important risk factors, while warranted, lies beyond the scope of this particular paper.

21. Focus the study on root causes of homicide in men, and remove the focus on comparisons between men and women. An unintended consequence of this study would be to defund initiatives focused on homicide and injury prevention in women and children, so this manuscript needs to be completely rewritten to prevent this sort of downstream policy consequence.

Our response:

With respect, we strongly refute the reviewer’s conclusion. First, at no point have we indicated that the paper is about root causes of homicide and the data we have collected are simply not suitable for this purpose. Second, we do not understand how identifying violence prevention as a priority based on good quality, but essentially descriptive, empirical data would detract from and lead to the defunding of any evidence-based violence prevention initiatives. On the contrary, we believe that violence prevention should always be informed by the most accurate epidemiological data and high-quality intervention studies. We do not see men and women as competing populations in terms of public health or the right to health.

We also believe that recognizing male homicide as a prevention priority will have beneficial consequences at a societal level including for men, women, male and female children and non-binary individuals. This is because reducing male homicide requires the adoption of broad-based approaches, which do not favour one group over another and are therefore likely to yield universal reductions in violence across groups.

22. The conclusions need to be much more data driven. This could include a focus on geographic differences and associated differences in policy, firearm usage and policy changes over time within each of the regions affected, and other such detailed analyses of the data that can be more helpful from a policy standpoint.

Our response:

Thanks for this comment. As implied in 20 above, the current analysis, which essentially establishes a starting point for a broad research agenda to explore male homicide, is not conducive to inferring data driven conclusions on geographic differences in homicide incidents due to the effects of specific interventions and policies. Nevertheless we have taken the reviewer’s suggestions on board as defining important areas for future research that could be informed by the data we have collected.

23. The authors must confirm that the data is publicly available based on the guidelines of the PLOS journals.

Our response:

The PloS guidelines state: “**authors to make all data necessary to replicate their study’s findings publicly available without restriction at the time of publication. When specific legal or ethical restrictions prohibit public sharing of a data set, authors must indicate how others may obtain access to the data.**”https://journals.plos.org/globalpublichealth/s/data-availability

In our submission we responded to the data availability query as follows:

“Availability of data used in the study would be subject to permission by the Health Research Ethics Committee and provincial authorities that approved the original study.”

We have revised our submission by appending the following text:

“This is a recently completed study and the dataset will initially be used for capacity development among the emerging researchers on study team. Thereafter access to a de-identified dataset is available upon reasonable request.  Requests should be sent to the corresponding author for consideration. A period of 24 months after publication of the main study results should elapse before requests are made, to allow the authors to publish sub-studies and further analyses.

Finally, regardless of the final decision, we appreciate the constructive comments and the opportunity to revise and improve our submission. However, it would be remiss of us not to recognise and address the anomalous response to our manuscript by reviewer 3.

Unlike the editorial queries and comments from reviewer 1 and reviewer 2, which were overwhelmingly favourable, reviewer 3 suggests a total reconceptualisation and reanalysis of our data. The root of this seems to be an underlying notion of populations competing for prevention efforts, in which men are somehow less deserving. We believe that as well as being anathema to public health and human rights, this is fundamentally not good science as it represents a bias against men – one that we encounter frequently.

We reiterate that prevention should always be informed by the most accurate epidemiological data and high-quality intervention studies and that recognizing male homicide as a prevention priority will have broad beneficial consequences at a societal level. We hope that this paper goes some way to foregrounding men as an important target population for violence prevention efforts and we would be grateful if *PLoS Global Public Health* supported these efforts through the publication of our study.

With thanks and regards

Richard Matzopoulos (on behalf of the authors)
