# Supplementary material for: South Africa’s male homicide epidemic hiding in plain sight: Exploring sex differences and patterns in homicide risk in a retrospective descriptive study of postmortem investigations
Source: PLOS Glob Public Health. 2023 Nov 22;3(11):e0002595. doi: 10.1371/journal.pgph.0002595 (PMC10664949; doi:10.1371/journal.pgph.0002595)
Supplement: Attachment — Submitted filename: Reviewer Comments PLOS Global Health South Africa Homicide.docx [file pgph.0002595.s004.docx]

Thank you for submitting this original study focused on understanding the burden of homicide in the South African context. This study retrospectively analyzed data from postmortem investigations, and focused on sex specific differences in rates of homicide. The conclusions of the study focus on the fact that men are significantly more likely to be victims of homicide than females, and the statistical methods focus on this as the main conclusions.

While the data represents a very interesting source of information, the conclusions need to be considerably refocused. The comparison between homicide in men and women while justified in theory by the data, does not really provide any helpful information that should educate future interventions. The focus on gender is a missed opportunity in terms of use of this data, and this paper would benefit from being focused more on the high rates of homicide in men and specific sources of homicide rooted in geography, sociodemographic characteristics other than gender, and a more data-driven conclusion. The authors seem to interject a lot of opinion in the study regarding poverty, inequality, firearm use, and alcohol use, but this is not really discussed in full detail in the study because of the focus on gender.

1. Focus the study on root causes of homicide in men, and remove the focus on comparisons between men and women. An unintended consequence of this study would be to defund initiatives focused on homicide and injury prevention in women and children, so this manuscript needs to be completely rewritten to prevent this sort of downstream policy consequence.
2. The conclusions need to be much more data driven. This could include a focus on geographic differences and associated differences in policy, firearm usage and policy changes over time within each of the regions affected, and other such detailed analyses of the data that can be more helpful from a policy standpoint.
3. The authors must confirm that the data is publicly available based on the guidelines of the PLOS journals.

Thank you for this submission. The authors must rewrite this study and ensure that it addresses the unintended consequences of highlighting rates of homicide as being a non-issue in women compared to men before additional consideration is given.
